# Flicker-assisted localization microscopy reveals altered mitochondrial architecture in hypertension

**DOI:** 10.1038/srep16875

**Published:** 2015-11-23

**Authors:** Susan Chalmers, Christopher D. Saunter, John M. Girkin, John G. McCarron

**Affiliations:** 1 Strathclyde Institute of Pharmacy & Biomedical Sciences, 161 Cathedral Street, University of Strathclyde, Glasgow G4 0RE, UK; 2 Department of Physics, Durham University, South Road, Durham DH1 3LE, UK

## Abstract

Mitochondrial morphology is central to normal physiology and disease development. However, in many live cells and tissues, complex mitochondrial structures exist and morphology has been difficult to quantify. We have measured the shape of electrically-discrete mitochondria, imaging them individually to restore detail hidden in clusters and demarcate functional boundaries. Stochastic “flickers” of mitochondrial membrane potential were visualized with a rapidly-partitioning fluorophore and the pixel-by-pixel covariance of spatio-temporal fluorescence changes analyzed. This Flicker-assisted Localization Microscopy (FaLM) requires only an epifluorescence microscope and sensitive camera. In vascular myocytes, the apparent variation in mitochondrial size was partly explained by densely-packed small mitochondria. In normotensive animals, mitochondria were small spheres or rods. In hypertension, mitochondria were larger, occupied more of the cell volume and were more densely clustered. FaLM provides a convenient tool for increased discrimination of mitochondrial architecture and has revealed mitochondrial alterations that may contribute to hypertension.

Mitochondria contribute to proliferation, migration, contraction, phenotype modulation and cell death by regulating signaling and energy requirements^1^,^2^. The shape, size, location and inter-connectivity of mitochondria is hypothesized to be central to the control of cell function and may enable the organelle to transmit or isolate localized rises in cytosolic Ca^2+^ concentration ([Ca^2+^]_c_) to regulate ATP supply and reactive oxygen species production^3^,^4^. In performing each of these functions, various arrangements of the organelle have been recognized. Small spheres, swollen spheres, straight rods, twisted rods, branched rods and loops, branched networks and even single continuous mitochondrial reticula have all been described^5^,^6^,^7^,^8^. The detailed structure of the organelle has been derived largely from high-quality imaging studies in cultured cells lines. Cultured cells are particularly amenable to imaging because the cells are thin in the axial direction and can be transfected to express unique, mitochondrially-targeted fluorophores, to give exceptional signal to noise for imaging mitochondrial structure. On the other hand, within freshly-isolated *ex vivo* cells or tissues much less is known about mitochondrial structure. Mitochondria in *ex vivo* cells are difficult to visualize when compared to cultured cells because the former can have a significant depth. As a result, there are multiple overlapping mitochondria in the axial direction, and, in the lateral direction, organelles may pack closely. Although viral transfection vectors and mouse transgenics have provided some successful tools, an additional difficulty in visualizing mitochondria arises from the challenges in targeting fluorophores exclusively to mitochondria in various freshly-isolated *ex vivo* cells. The lack of exclusive fluorophore labelling, as can be achieved in cultured cells, limits the signal-to-noise ratio available. When mitochondria in freshly-isolated *ex vivo* cells are viewed fluorescently, each of these features (axial overlap, lateral packing, lack of exclusive fluorophore targeting) create numerous merged fluorescence point sources at different depths that may entirely obscure mitochondrial structure. Whether or not the arrangements of the organelle seen in cultured cells occur *in vivo* is uncertain and the functional significance of whatever arrangements exist is unknown.

A first step in understanding the functional significance of mitochondrial arrangement in freshly-isolated *ex vivo* cells, and how drugs or disease modify them, requires a characterization of the structure of the organelles in live cell imaging. Optical sectioning imaging methods, such as confocal microscopy, may not provide sufficient resolution to determine detailed structure of mitochondria in freshly-isolated *ex vivo* cells, where multiple membranes in close proximity creates optical confusion. Super-resolution techniques^9^,^10^,^11^,^12^,^13^,^14^ can be challenging for live cell work, although some elegant recordings of dynamic mitochondria have been made over a few minutes using live-cell stochastic optical reconstruction microscopy^15^. Super-resolution or electron microscopy (EM)^16^ techniques, however, may not always unambiguously reveal the extent of the electrically-continuous inner mitochondrial membrane. Detailed insights into the structure of individual mitochondria at fixed points in time have been provided by EM^3^ but cannot easily resolve the mitochondrial complement of an entire cell. There is now a strong drive to provide information on the dynamic changes or functional connectivity of mitochondria. We have developed a technique to discriminate the structure of individual, electrically-discrete, polarized mitochondria in live, freshly-isolated *ex vivo* cells or tissues. Our approach is rapid, compatible with conventional widefield fluorescence imaging, and quantifies the shape, size and location of mitochondria throughout the cell and even within dense organelle clusters.

Transient openings of the mitochondrial permeability transition pore (mPTP)^17^,^18^, cause rapid changes of the membrane potential (ΔΨ_m_) of individual mitochondria and can be photo-induced^19^. These ΔΨ_m_ changes can be visualized using a rapidly-repartitioning cationic fluorophore such as TMRE, producing localized flickers of fluorescence that demarcate individual, electrically-discrete mitochondria^20^,^21^ that have previously been used to show mitochondrial arrangements^22^,^23^ and covariance calculation from temporally differentiated images^23^ has been used to provide measurements of mitochondria in cultured cells. We have extended this analysis by including a critical ‘all-on’ step prior to calculating the covariance from temporally differentiated images which rapidly corrects for bleaching, drift and variations in light intensity and the analysis is suitable for use in intact tissue and fully differentiated *ex vivo* cells. Drawing inspiration from PALM/BaLM super-resolution techniques^11^,^12^, we use these flicker events to extract structural information, facilitating a more detailed analysis of the fluorescence images. Extracting information from these flickers differs from the localization of photo-activation, bleaching or blinking in PALM/BaLM because the flickering objects are not single molecule emitters with a well-defined point-spread function but extended organelles of unknown shape. None-the-less, the size and shape of the mitochondria can be rapidly determined to the conventional resolution limit from the spatio-temporal covariance of the derived flickering fluorescence signals and is the approach described herein. The positional relationships between organelles within the irregular environment of the cell can then be addressed using Voronoi diagrams to quantify relative mitochondrial clustering.

We show, in freshly-isolated *ex vivo* resistance artery smooth muscle cells, the wide variation in apparent size of mitochondria is partially explained by varying numbers of small organelles within tightly-packed organelle clusters. We also show in hypertension, a condition associated with increased oxidative stress, that the individual mitochondrion size and the mitochondrial cluster density increases in the smooth muscle cells. Flicker-assisted localization microscopy (FaLM) thus provides a convenient method to compare mitochondrial size, shape and position and the resulting data suggests that changes in mitochondrial structure contribute to the altered vascular performance in hypertension.

## Results

### Flicker-assisted Localization Microscopy of live-cell mitochondrial structure

**Figure 1:**
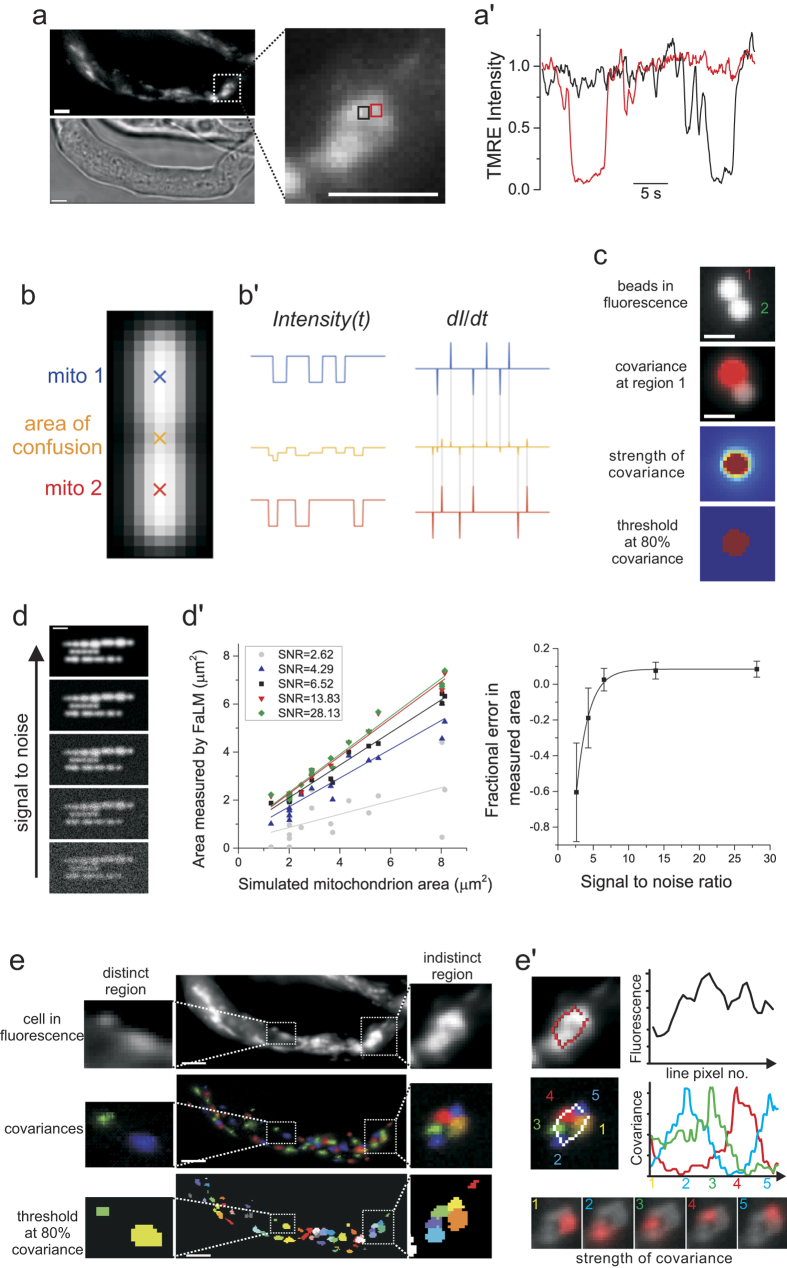
Mitochondrial boundaries that are unclear in fluorescence images are resolved by FaLM. (**a**) Epifluorescence images of mitochondria in freshly-isolated *ex vivo* smooth muscle cells (top and expanded: TMRE; lower: brightfield) shows visually-indiscriminate regions that display independent, transient changes in fluorescence due to ΔΨ_m_ flickering (**a′**: fluorescence intensity of two neighboring 0.2 μm^2^ regions shown in **a**), scale bars 10 μm. (**b**) A model fluorescent image of two mitochondria positioned close enough to share an “area of confusion”, where the boundary of each cannot be distinguished, produce fluorescence patterns (**b′**) in the center-points of each mitochondrion which overlap but become distinct when differentiated (*dI/dt*). (**c**) FaLM resolves individual fluorescent beads whose fluorescence is induced to fluctuate by transient illumination from a 2 μm-diameter laser source. Analysis of the covariance in fluorescence intensity changes over time (covariance in red overlaid on image of starting fluorescence, second top) shows strong covariance only of the “flickering” bead (third). A threshold of 80% covariance returns an object of approximately the expected size (bottom). Scale bars 2 μm. (**d**) The ability of FaLM to detect flickering objects was assessed by creating model image stacks of 18 stochastically-flickering fluorescent objects blurred by a realistic point spread function plus simulated photobleaching, read-noise and photon-noise, resulting in 5 image stacks with increasing signal to noise ratio (SNR) from 28.13 (top) to 13.83, 6.52, 4.29 and 2.62 (bottom); scale bar 2 μm. (**d′**) FaLM accurately reported the object sizes at all SNR > 4.3 (left plot); the fractional error in mean object size ± s.d. (calculated area/actual area, *n* = 6 objects of area 2 μm^2^) decreases exponentially with increasing SNR (right plot). (**e**) Epifluorescence images containing both indistinct and distinct regions of mitochondrial fluorescence (top) are converted by FaLM, resolving the spatio-temporal covariances of fluorescence intensity due to flickers (middle), from which a threshold of 80% covariance defines mitochondrial boundaries (bottom). This process clearly resolves five distinct mitochondria within the “indistinct” expanded image of **e**. Line profiles of intensity show the resolution of five objects in the covariance of fluorescence changes that are unclear in standard fluorescence intensity (**e′**). Scale bars 10 μm.

Mitochondria display a range of sizes (~0.5–10 μm) in standard epifluorescence imaging and can appear as visually-indistinct regions that could comprise large networks or various sizes of tightly-packed organelles (Fig. 1a). In live, freshly-isolated vascular smooth muscle cells, high resolution 3-dimensional z-stack images of mitochondria (150 × 150 × 100 nm voxel), deconvolved to maximize image quality, also show the organelles both as small ovoid shapes (largely orientated with their long-axis in line with that of the cell, Supplementary Video 1) and as regions that could comprise either networks or multiple individual organelles. To definitively identify electrically-contiguous mitochondria, transient ΔΨ_m_ depolarizations were induced by mild photo-activation of the mPTP. The cationic fluorophore TMRE rapidly redistributes in a Nernstian-manner according to ΔΨ_m_, providing an optical readout of these electrical events, referred to as “flickers”. Flickers began within 2–3 min of illumination and reversible ΔΨ_m_ changes occurred during the first 10 min; analysis was limited to this period. Neighboring regions were often observed to flicker completely independently (Fig. 1a′ and Supplementary Video 2) demarcating individual mitochondria. To extract individual organelle identities (sizes, shapes and positions) Flicker-assisted Localization Microscopy (FaLM) was developed (Fig. 1b and in more depth in Supplementary Fig. 1), which resolves individual organelles even when their images share pixels due to dense packing or vertical overlap. Two mitochondria whose fluorescence images overlap result in an area of confusion (AoC) where the boundaries of the organelles are ambiguous (Fig. 1b); plotting fluorescence intensity versus time for an individual pixel near the center of each candidate mitochondrion (mito 1 and 2) reveals two uncorrelated flicker signals, whereas in the AoC the signals are mixed and thus not easily separable. The derivative of these signals, *dI/dt* however, localizes in time the depolarization periods to two brief transient spikes whose duration is determined by the near-instantaneous time required to de- and re-polarize. These signals form unique fingerprints of each organelle’s identity and remain clearly separate, even within the AoC, and thus present a suitable basis for discrimination. Supplementary Video 2 shows the flickers of TMRE fluorescence alongside the *dI*/*dt*, which shows the onset (red) and end (blue) of each flicker event. The covariance between the *dI*/*dt* of a candidate mitochondrial center and those of neighboring pixels directly recovers the image of that organelle. The full algorithm and individual steps are described in Methods and shown in Supplementary Fig. 1. Briefly, each image in the raw stack is subtracted from a corresponding time-localized maximum fluorescence projection, corresponding to all mitochondria being fully polarized. The resulting *Delta* stack is then differentiated with respect to time to extract the onset, and exit, of depolarizations (*dI*/*dt*). The spatio-temporal covariance of this differentiated signal is used to define the spatial extent of each mitochondrion. These mitochondria can then be recombined to provide an image of all individual mitochondria in the cell and spatial measurements (length, width and positioning relative to other mitochondria) obtained.

This technique was validated using fluorescent beads (4 μm diameter, FocalCheck slide #1, row A3, Life Technologies) whose fluorescence intensity was transiently increased by a highly-localized laser beam (2 μm diameter illumination spot in the sample plane, 0.5 ms duration pulses with 600 ms between), coupled in through the excitation lightpath of the microscope. These transient increases in fluorescence were measured as a localized signal covariance in FaLM that recovered the image of only the stimulated area of the bead and not others nearby (Fig. 1c). Applying a threshold to the spatio-temporal covariance image of 80% (i.e. including only pixels with covariance values of > +0.4, on a scale of −1 to +1) produced an object with a diameter of 2.6 μm (for our 2 μm localized stimulation, a reasonable discrepancy in diameter considering that we are resolving the diffracted image of a sphere). To further characterize the ability of FaLM to detect flickering mitochondria, we created a set of model image stacks of stochastically-flickering fluorescent objects. The images were blurred using a Gaussian point spread function and subjected to simulated bleaching, photon-noise and read-noise. Five image stacks of increasing signal to noise ratio were created (ranging from 2.62 to 28.13 at the fluorescent object, still images shown in Fig. 1d) and processed using FaLM to measure the XY areas of the objects. The FaLM-measured areas accurately reported the actual areas of simulated flickering mitochondria of a range of sizes for the image stacks with signal to noise ratios >4.3 (Fig. 1d′, left-hand panel). There was an exponential increase in under-reporting measurement error as signal to noise decreased below ~5 (Fig. 1d′, right-hand panel); however this value is well below the signal to noise ratios that we observe experimentally. The experimental sample measurements of signal to noise of TMRE fluorescence in our imaging system range from 22 to 70.

Image analysis using FaLM resolved multiple, separate mitochondria where single, large organelles might otherwise be assumed. Figure 1e shows a region of TMRE fluorescence that appeared to arise from a mitochondrial network with no clear boundaries, however, five separate regions of distinct *dI/dt* covariance allowed discrimination of five electrically-discontinuous mitochondria (Fig. 1e′).

**Figure 2:**
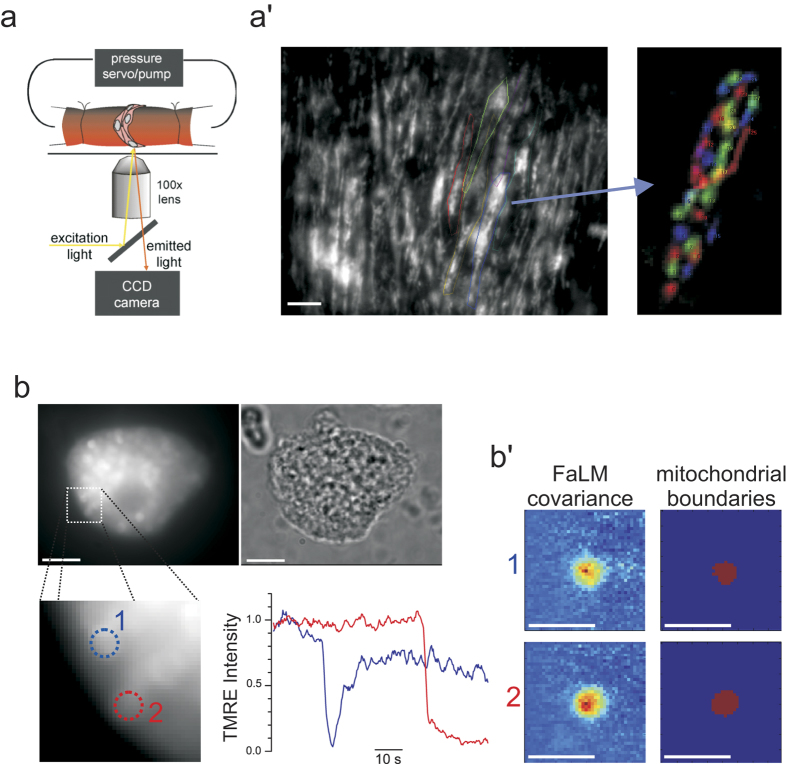
FaLM resolves oblate-spheroid mitochondria in intact, pressurized resistance arteries and spherical mitochondria in freshly-isolated hepatocytes. (**a**) Intact posterior cerebral resistance arteries (~250 μm diameter) were maintained *ex-vivo* at physiological pressure (40 mmHg) and mitochondrial TMRE imaged on an inverted epifluorescence microscope. (**a’**) FaLM resolves individual mitochondria (FaLM covariance in color, right) from indistinct epifluorescence images (TMRE fluorescence, left, annotated with estimated cell boundaries for ease of viewing). (**b**) Hepatocytes freshly-isolated from rat liver contain many spherical mitochondria (TMRE, left; brightfield, right) that also display spatial- and temporally-distinct flickers that FaLM can utilize to define boundaries of individual, electrically-distinct mitochondria, **b’**. scale bars 10 μm (a’,b), or 5 um (b’).

FaLM is also compatible with imaging mitochondrial morphologies in intact tissue. In *ex-vivo* pressurized resistance arteries, mitochondria imaged with FaLM have a median area of 2.08 μm^2^ with similar morphologies to those observed in freshly-isolated smooth muscle cells (Fig. 2a). FaLM can be applied to resolve mitochondrial structure in different cell types. In freshly-isolated hepatocytes imaged by FaLM, mitochondria display multiple spherical structures (Fig. 2b).

**Figure 3:**
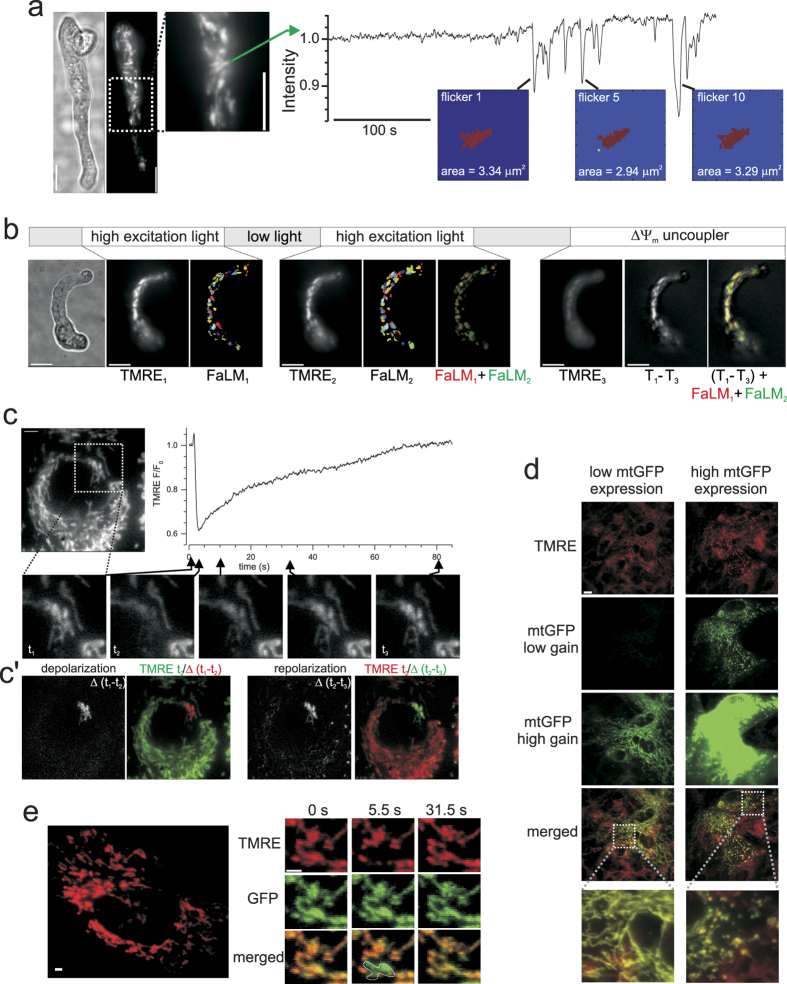
FaLM recovers mitochondrial architecture consistently and repeatedly, for small or large-networked mitochondria, and without the need for mt-GFP expression. (**a**) Mitochondria within a freshly-isolated *ex vivo* myocyte (upper: brightfield, lower: TMRE) displayed repeated, transient ΔΨ_m_ oscillations after ~3 min (graph shows fluorescence fluctuations for one example mitochondrion, green arrow). Each flicker was analyzed individually by FaLM and the resultant dimensions for the first, fifth and tenth flicker of the same mitochondrion show no change in morphology or dimensions. Scale bars 10 μm. (**b**) Modification of excitation light source intensity (upper bar) allows bursts of flickering at various times from which FaLM detected reproducible mitochondrial architectures (colored panels) from TMRE fluorescence (black and white panels, all at same intensity range). FaLM-detected objects from the first and second flicker periods are very similar in size and shape (overlaid in FaLM_1_ + FaLM_2_ panel). Subsequent depolarization of all mitochondria following CCCP (1 μM) results in a loss of TMRE fluorescence (TMRE_3_). Subtracting image TMRE_3_ from TMRE_1_ reveals all pixels associated with mitochondrial TMRE fluorescence and that FaLM detected all polarized mitochondria. Scale bars 10 μm. (**c**) Myocytes maintained in cell culture often contain large, network-like mitochondria that show synchronous decrease and restoration of TMRE fluorescence with no morphological alterations. Image subtraction, **c’**, shows the regions that de- and re-polarized in isolation (black and white frames) and then relative to the rest of the cell (colored frames). Scale bar 5 μm. (**d**) Cultured myocytes display various levels of mitochondrially-targeted GFP (mtGFP) expression and hence fluorescence was compared at low and high gain. Overlay of mtGFP and TMRE fluorescence (lower panels) shows that high mtGFP expression is associated with a more punctate mitochondrial morphology. Scale bar 10 μm. (**e**) The morphology of individual mitochondria can be recovered even when the organelles are highly-motile by expressing mtGFP (green), loading with TMRE (red) and merging image pairs during flicker events (lower panels). Scale bar 2 μm.

Care was taken to ensure mild photo-activation, resulting in a slow onset of flickers and enabling comparison of Ψ_spatial_ (the static fluorescence of a mitochondrion) obtained from the first time a mitochondrion flickered with Ψ_spatial_ obtained from later flickers. Figure 3a shows that there is no difference in the size of individual mitochondria measured from their first and subsequent flickers. Repeated “bursts” of flickering could be obtained by attenuating the excitation light intensity during the course of an experiment as shown in Fig 3b. In this example, two 3 min spells of increased excitation light intensity, with a 5 min rest period between, produced flickers that were analyzed independently using FaLM. The two sets of mitochondrial objects (FaLM_1_ and FaLM_2_) obtained were highly colocalized (Fig. 3b, FaLM_1_ + FaLM_2_, 91% colocalization), indicating that repeated measures of mitochondrial architecture are possibly. Extended periods of photo-activated mitochondrial flickering is likely to cause cellular damage, hence the minimum duration necessary for the preparation should be used if prolonged cell function is required. Figure 3b (panel TMRE_3_) shows subsequent depolarization of the entire mitochondrial complement of the cell with the protonophore CCCP (1 μM) to reveal all organelles. Subtracting the “after CCCP” image from the starting image (panel T_1_-T_3_) shows all of the cell’s polarized mitochondria, which was very well colocalized with the mitochondrial regions revealed by the preceding FaLM (98.8% colocalization) indicating that all polarized mitochondria were detected by FaLM.

Mitochondrial depolarization itself *can* cause alterations in the morphology of the organelle^7^,^24^,^25^, however we did not observe flicker-induced changes in mitochondrial morphology–indeed some large, networked mitochondria could be seen to de- and re-polarize whilst maintaining their complex structure (Fig. 3c, see also^23^). On the other hand, these large mitochondrial structures *can* become more punctate in cells which transiently expressed mitochondrially-targeted GFP (mt-GFP) at high levels (Fig. 3d; 87.5% of cells expressing high levels of mt-GFP contained mostly punctate mitochondria, *N* = 8, compared to only 12% of cells expressing low levels of mt-GFP, *N* = 25; proportions significantly different by two-tailed Z-test for two populations, *Z* = 4.044, *p* < 0.001). If expressed at low- to moderate-levels however, mt-GFP can be used in combination with TMRE to resolve the shape and position of individual, electrically-distinct mitochondria–even within a highly-motile organelle population - by subtracting flickering TMRE images from time-matched constant mt-GFP images (Fig. 3e).

### Mitochondrial structure alterations in a model of hypertension

**Figure 4:**
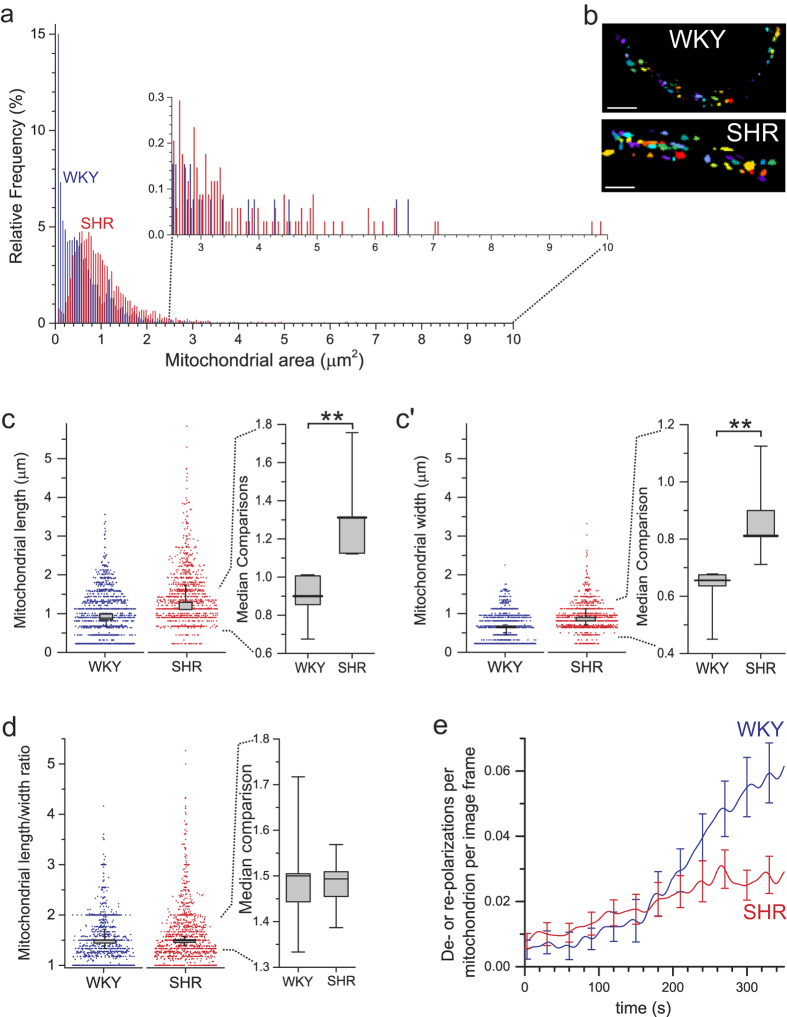
Mitochondrial dimensions and rate of flickering in hypertension. (**a,b**) Mitochondria in freshly-isolated *ex vivo* myocytes from both hypertensive (SHR, red) and normotensive control (WKY, blue) rats displayed a range of sizes that was shifted to larger areas in hypertension, scale bars (**b**) 5 μm. (**c**) Mitochondria in SHR were longer and wider than those in WKY (left-hand panels in **c** and **c’** show individual measurements of each mitochondrion with summarized data of median values for each animal superimposed, these median values alone are shown in the expanded-scale panels at right of **c** and **c’**; median–thick lines, IQR–grey boxes, and max/min values - whiskers, **p < 0.01, *N* = 5 animals). (**d**) The mitochondrial length:width ratio, however, was the same in SHR and WKY (left panel shows individual ratios for each mitochondrion with summarized median values for each animal superimposed, these median values alone are shown in the expanded-scale right-hand panel; median values–thick lines, IQR–grey boxes, and max/min values-whiskers, *N* = 5 animals, p = 0.837). (**e**) Quantification of flicker events (ΔΨ_m_ de- or re-polarizations) in each imaging frame during FaLM imaging in myocytes from SHR (red) and WKY (blue) rats, raw data smoothed by 30-point FFT filter (hence over 10 s window), error bars are standard deviation of raw data.

Mitochondrial function and motility are altered in various models of vascular disease^26^,^27^, though most studies have been in cultured cells. Whether or not changes exist *in vivo* is uncertain^28^. As a first application of FaLM we examined whether mitochondrial size or position differed within freshly-isolated *ex vivo* vascular smooth muscle from the spontaneously hypertensive rat strain (SHR) compared to normotensive control Wistar-Kyoto (WKY). Single smooth muscle cells, freshly-isolated from resistance-sized posterior cerebral arteries from age-matched SHR and WKY rats, were loaded with TMRE (62.5 nM) and image stacks of flickers captured and analyzed as described. Figure 4 shows the range of sizes of all of the mitochondria in SHR and WKY smooth muscle (Fig. 4a,b). The majority of mitochondria in WKY were very small (median area of 0.46 μm^2^ with a large interquartile range, IQR, of 0.131–0.789 μm^2^ but a mode, most common size, of 0.051 μm^2^) compared to those in SHR (median 0.91 μm^2^, IQR = 0.536−1.28 μm^2^ and mode 0.62 μm^2^; for *N* = 5 animals from each strain, there was a significant difference between strains as determined by two-sample Kolmogorov-Smirnov comparison of the median mitochondrial sizes for each animal, *D* = 1, *Z* = 1.58, *p* = 0.00794). There was few mitochondria observed in SHR with the minimum area observable under these conditions (one pixel = 0.0506 μm^2^) whereas 15% of mitochondria in WKY were of this size or smaller. On the other hand, there were very few large mitochondria in WKY compared to SHR (Fig. 4a inset), the largest 5% of mitochondria had an area >1.7 μm^2^ in WKY compared to >2.3 μm^2^ in SHR. Thus there was a clear difference in mitochondrial size between normotensive and hypertensive smooth muscle.

Total cell area was not different between the two groups. Cells from WKY had a median area of 437 μm^2^ (IQR = 361–501 μm^2^) compared to 451 μm^2^ (IQR = 357–515 μm^2^) in SHR (no significant difference between strains as determined by two-sample Kolmogorov-Smirnov test, *D* = 0.4, *Z* = 0.632, *p* = 0.873; *N* = 5 animals from each strain). There were the same number of mitochondria per unit cell area in SHR and WKY (median of 0.165 mitochondria per μm^2^ in SHR, IQR = 0.122-0.189 mitochondria/μm^2^, compared to 0.125 in WKY, IQR = 0.087-0.177 mitochondria/μm^2^; no significant difference between strains as determined by two-sample Kolmogorov-Smirnov test, *D* = 0.4, *Z* = 0.632, *p* = 0.837; *N* = 5 animals from each strain). There was, however, a greater proportion of the cell occupied by mitochondria in SHR than WKY (median of 19.7% in SHR, IQR = 17.8–23.4%, compared to 7.0% in WKY, IQR = 5.9–12.1%; significant difference between strains as determined by two-sample Kolmogorov-Smirnov test, *D* = 1, *Z* = 1.58, *p* = 0.00794; *N* = 5 animals from each strain).

In addition to the changes in mitochondrial volume, the relative dimensions of the organelle may alter due to disease. Mitochondria in smooth muscle from hypertensive animals were both longer and wider than those from the normotensive control (Fig. 4c; median length of mitochondria was 1.31 μm in SHR, IQR = 1.13–1.53 μm, compared to 0.9 μm in WKY, IQR = 0.77–1.01 μm, two-sample Kolmogorov-Smirnov test, *D* = 1, *Z* = 1.58, *p* = 0.00794; *N* = 5 animals from each strain; median width was 0.81 μm in SHR, IQR = 0.71–1.01 μm, compared to 0.66 μm in WKY, IQR = 0.54–0.68 μm, two-sample Kolmogorov-Smirnov test, *D* = 1, *Z* = 1.58, *p* = 0.00794; *N* = 5 animals from each strain). The median length:width ratio of mitochondria did not alter, however (Fig. 4d; 1.49 in SHR, IQR = 1.42-1.54, compared to 1.5 in WKY, IQR = 1.39–1.61, two-sample Kolmogorov-Smirnov test, *D* = 0.2, *Z* = 0.32, *p* = 1; *N* = 5 animals from each strain). In addition to spatial information, FaLM enables a functional analysis of the rate of flickering in different samples. We examined the number of flicker events per mitochondrion during the flicker process, defining “events” as |*dD*′*/dt*|>0.075/s, where *D*′ is the *delta* signal normalized to the *all on* signal and smoothed with a 7th order, 21 frame Savitzky-Golay filter^29^. Interestingly, mitochondria in SHR myocytes initially flickered at a slightly higher rate than those in WKY, but then after ~200 s there was a sharp rise in the rate of flickering of mitochondria in WKY but not SHR myocytes, suggesting differences in mPTP kinetics (Fig. 4e).

**Figure 5:**
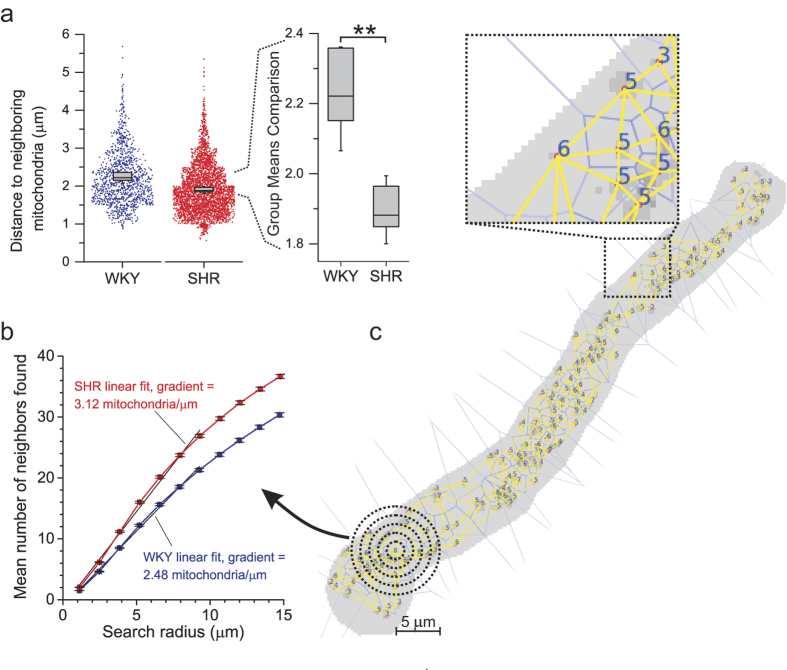
Mitochondrial clustering in hypertension. (**a**) Mean distance between neighbors was less in SHR than WKY (left panel shows individual distances between each pair of neighboring mitochondria with summarized mean values for each animal superimposed, these mean values alone are shown in the expanded-scale right-hand panel; mean–thick lines, IQR–grey boxes, and max/min values–whiskers, **p < 0.01, *N* = 5 animals). The number of neighbors and distances between each neighbor were calculated from Voronoi plots (e.g. **c**) of the centerpoints of each mitochondrion identified by FaLM; close-up shows the inter-mitochondrial boundaries in blue, the shortest distance between centers in yellow and the number of neighbors written at each centerpoint. (**b**) Mitochondrial density was also calculated by counting the number of mitochondria that exist in areas of expanding search radius (e.g. dotted circles in **c**) for each mitochondrion (mean ± SEM for SHR, red, and WKY, blue; linear fit shown over first 10 μm).

We next determined the distribution of mitochondria through the cell by examining the relative positioning and clustering of the organelles using Voronoi plots of the centerpoints of each mitochondrion (described in Methods, example shown in Fig. 5c). Voronoi plots provide a quantitative method to determine the number of neighbors each mitochondrion has and the distances between neighbors. In hypertension each mitochondrion had the same average number of neighbors as the WKY controls, but the inter-mitochondrial distance measured between centerpoints of neighboring mitochondrial pairs decreased. Mitochondria in SHR had 4.68 ± 0.13 neighbors compared to 4.53 ± 0.05 in WKY (mean ± SEM neighbors compared by two-sample t-test of means with Welch correction for unequal variance, *t* = 1.999, *DF* = 7.94, *p* = 0.08, *N* = 5 animals for each strain). The mean distance between neighboring mitochondria was 1.90 ± 0.04 μm in SHR compared to 2.23 ± 0.06 μm in WKY (mean ± SEM neighbors compared by two-sample t-test of means with Welch correction for unequal variance, *t* = −4.898, *DF* = 6.69, *p* = 0.002, *N* = 5 animals for each strain; Fig. 5a). As an alternative method to analyze mitochondrial clustering, the number of neighbors that each mitochondrion had was counted using an expanding search radius (illustrated in Fig. 5c). This analysis showed a nearly linear relationship between increasing radius and the number of mitochondria encountered for both populations when the search radius was <10 μm. The relationship then became less linear due to the limiting effects conferred by cell boundaries (Fig. 5b). At all radii (below 10 μm) mitochondria in hypertensive smooth muscle had more neighbors than in normotensive control, with an average of 3.12 mitochondria encountered per μm in SHR compared to 2.48 mitochondria per μm in WKY. Thus mitochondria within smooth muscle from hypertensive animals were more tightly clustered together than those in smooth muscle from normotensive animals. These changes in mitochondrial architecture and relative positioning may contribute to the alterations in smooth muscle physiology observed in hypertension.

## Discussion

Mitochondrial structure is central to the organelle’s control of virtually every physiological and pathological process. Mitochondria may be closely-packed, small units or interconnected networks and can be highly motile, rapidly-reshaping structures that exist in a complex, 3D arrangement. The organelle’s architecture may contribute to cell function by facilitating or restricting signal or metabolite propagation (e.g. through a reticular network versus punctate mitochondria) and their relative cellular positioning determines interactions with other organelles (e.g. SR) and localized signaling events. The density of organelles and diversity of arrangements, however, have made imaging an entire mitochondrial population in live cells and tissue a challenging task.

Taking inspiration from super-resolution techniques, we have developed a method that defines the functional boundaries of electrically-discrete individual mitochondria that cannot be resolved conventionally. Our method (FaLM) rapidly defines the size, shape, position and density of the whole mitochondrial complement of a cell. There are differences in our approach from super-resolution techniques. Whereas uncorrelated blinking denotes separate molecules in BaLM, correlated fluorescence flickering in mitochondria denotes the extent of an electrically-connected (and hence functional) organelle in FaLM and requires fundamentally different analyses from PALM/BaLM.

Super-resolution techniques in which the stochastic photoactivation, bleach and blink events of single fluorescent molecules enable sub-diffraction limit resolution have been effective in resolving organelle structure. These techniques, however, do not determine *functional* boundaries and are often difficult to apply in living cells. Individual mitochondria are defined as having an electrically-continuous inner mitochondrial membrane and in FaLM we used the unique electrical signature of each mitochondrion to identify the organelles’ structures. The electrical signature arises from the transient, stochastic opening of the mPTP to cause a transient loss of ΔΨ_m_ that can be observed optically using rapidly-repartitioning ΔΨ_m_-sensitive fluorophores. FaLM rapidly and conveniently restores resolution of each functionally-separate mitochondrion, allowing the determination of each mitochondrion’s structure in live cells. FaLM can discriminate multiple, tightly-packed organelles (Fig. 1) and unambiguously determine the spatial-extent of large networked mitochondria (i.e. with an interconnected inner mitochondrial membrane, Fig. 3c). No knowledge of the microscope point spread function (PSF) is required and, significantly, the method works even when there is significant overlap of PSFs. FaLM accommodates non-uniform backgrounds and slow drifts in in fluorescence intensity (features which may cause difficulties in some other approaches) to determine the structural features of all individual polarized mitochondria in an otherwise unresolvable image. FaLM is not a super-resolution technique in the conventional sense, however it does provide a functional resolution that may be challenging for super-resolution or even electron microscopy to achieve (i.e. the ability to unambiguously show that a small mitochondrion, which may otherwise be obscured within a large cluster, is electrically-, and hence functionally-, discrete). FaLM thus restores the conventional resolution limit where it is lost to dense organelle crowding.

Mitochondria are commonly described as forming a dynamic, interconnected network^7^,^10^,^30^,^31^,^32^,^33^, although the vast majority of research has been carried out on single cells maintained in cell culture conditions–for justifiable reasons of optical clarity and access to genetic-manipulation techniques. Altered mitochondrial arrangements and shape occur in various cultured cell models of disease^2^,^32^,^34^,^35^,^36^. Changes in the architecture of the mitochondrial network also occur in cholesterol-overload of cultured vascular smooth muscle^37^ or cigarette smoke exposure of cultured airway smooth muscle^38^. We have shown previously that mitochondrial morphology and architecture changes rapidly (within 1-2 days) as smooth muscle cells switch from a non-proliferative *ex vivo* phenotype to a proliferative phenotype in cell culture conditions^26^. Yet, in freshly-isolated *ex vivo* smooth muscle cells, it remains unclear to what extent mitochondria form a network, largely due to the difficulties in rapidly transfecting targeted fluorescent proteins combined with the cells complex 3D cellular morphology. Previous epifluorescence studies in live, freshly-isolated *ex vivo* smooth muscle have shown regions that *appear* as larger network areas and multiple electrically-discontinuous organelles^21^,^39^. EM has been used to reveal mitochondrial structure and does allow distinction of individual mitochondria (and elegant reconstruction of their ultrastructure)^3^,^16^,^40^, but a sub-population frozen in time. Single EM sections of freshly-isolated *ex vivo* smooth muscle appear to show small, fairly round, individual (not networked) mitochondria of ~0.2–0.8 μm diameter (e.g.^41^). The organelle’s size may vary significantly both within and among cell types in other EM studies (0.2–10 μm length)^31^,^33^,^42^ and in disease (1–10 μm diameter)^7^,^32^,^34^,^35^,^36^, presumably with consequences for signaling and cell performance, though the physiological significance of the various arrangements remain unclear^28^. Photo-activation of mitochondrially-targeted GFP (mtPA-GFP) has provided elegant visualization of the processes of mitochondrial fusion and fission^43^,^44^,^45^ but cannot provide information about all of the mitochondria within a cell. FaLM enables discrimination of the dimensions of the entire mitochondrial complement of a cell, can be carried out in cells that are hard to transfect and allows comparisons in animal models of disease (without requiring interbreeding with mtPA-GFP animals).

Using FaLM we have shown that mitochondria are larger (Fig. 4a,b) in resistance artery smooth muscle from a hypertensive rat model when compared to normotensive controls.

The increased mitochondrial size in hypertension revealed by FaLM may be due to a shift in the balance of mitochondrial fusion and fission towards the former. In support, increased levels and activity of mitochondrial fusion proteins and decreased fission proteins have been observed previously in cardiomyocytes from hypertensive SHR compared to normotensive WKY rats^45^. Decreasing mitochondrial fission with Mdivi-1 (which inhibits the activity of the outer mitochondrial membrane fission protein Drp1) or P110 (which disrupts the interaction between Drp1 and the mitochondrial-docking protein Fis1) is protective, reducing the severity of myocardial infarction caused by ischemic reperfusion^46^,^47^.

Hypertension is associated with decreased endothelial function and hence decreased production of nitric oxide (NO^48^, although whether or not alterations occur, and at what age they occur, in SHR are matters for debate^49^). Interestingly, in neurons, NO has been shown to cause S-nitrosylation of Drp1 to increase mitochondrial-fission activity^50^. On the other hand, proteins associated with mitochondrial fusion (Mfn1/2 and OPA1) were decreased and mitochondrial fission proteins (Drp1 and Fis1) were increased in rat aorta after 3 days L-NAME treatment to inhibit NO production^51^. NO can also repress the activity of Drp1 via cGMP/protein-kinase G mediated phosphorylation of the fission protein, which allows mitochondria to elongate and skeletal myogenic precursor cells to differentiate^52^. It remains to be determined whether or not alterations in endothelial NO production (or compensatory mechanisms such as the activation of inducible NO synthase) in hypertension contribute to alterations in mitochondrial morphology and function.

We have also shown here that mitochondria in resistance artery smooth muscle from hypertensive animals are clustered together more tightly than those in control (Fig. 5). When examining the relative positions of the centerpoints of each mitochondrion we found that in hypertension the organelles are closer together. We did not see a difference in cell size between the two groups. Earlier studies reporting differences in size, examined different blood vessels at an earlier age and found an increased cell size in SHR e.g.^53^. As the cells themselves were the same size in the resistance arteries from 250–300 g/13,14 week old animals examined in this study, but the mitochondrial complement occupied 20% of the cell volume in hypertension compared to 9% in control, it may be that the mitochondria in hypertensive smooth muscle *had* to be closer together. This increased clustering may influence a number of cellular processes–such as the formation of localized hotspots of production of ATP, ROS, glutathione or calcium cycling; or altered interactions with the sarcoplasmic reticulum, plasma membrane or nuclear processes.

In summary, FaLM is a novel, accessible method to unambiguously determine the size of individual mitochondria in live, *ex vivo* cells and tissues that provides detailed structural information with standard fluorescence imaging systems. FaLM opens possibilities for examining mitochondrial structures in a wide variety of cells and tissues, including transgenic animals or human tissue in clinical studies without a requirement for expression of fluorophores. FaLM uses the localized covariance of the unique spatio-temporal pattern of stochastic, transient flickers of ΔΨ_m_ of individual mitochondria to recover the image of each electrically-discontinuous mitochondrion within an otherwise optically complex cellular image. This functional resolution of organelle boundaries in clusters of visually-inseparable mitochondria has revealed previously-unrecognized increases in mitochondrial size and clustering in hypertension.

## Materials and Methods

### Animal maintenance and euthanasia

All experiments were carried out on tissue collected immediately after euthanasia (as described below) of animals not subject to any other treatments. Animal maintenance and euthanasia were in accordance with UK regulations (Animals (Scientific Procedures) Act 1986, revised under European Directive 2010/63/EU) and were approved by the University of Strathclyde Animal Welfare and Ethical Review Board. 5 male Wistar Kyoto, WKY, and 5 male Spontaneously Hypertensive, SHR rats (~250–300 g, 13–14 weeks of age, allowed *ad libitum* access to food and water with a reversed 12-hour light/dark cycle) were euthanized by trained technicians with an intraperitoneal overdose of sodium pentobarbital (Euthatal, 200 mg/kg) prior to rapid removal of the brain into MOPS-buffered saline (145 mM NaCl, 2 mM 3-(N-morpholino) propanesulfonic acid), 1.2 mM NaH_2_PO_4_, 4.7 mM KCl, 1.17 mM MgCl_2_, 5 mM glucose, 0.02 mM EDTA and 2 mM CaCl_2_, pH 7.4) and micro-dissection to clean the required arteries from adjacent tissue.

### Cell isolation and intact artery preparation

Smooth muscle cells from the superior cerebellar and posterior cerebral arteries were prepared as previously described^26^. Cells were loaded for 20–30 min with, and imaged in, TMRE (62.5 nM, in 55 mM NaCl, 80 mM Na-glutamate, 6 mM KCl, 1 mM MgCl_2_, 10 mM glucose, 10 mM HEPES, 0.2 mM EDTA and 0.1 mM CaCl_2_, pH 7.3, 37 °C) on an inverted epifluorescence microscope (Nikon TE2000-U with 100× 1.3 NA S-Fluor). Alternatively, the suspension of freshly-isolated cells was diluted 1:9 in 50:50 Ham F-12 and Waymouth MB752 media supplemented with 10% fetal bovine serum, 0.1 U/mL penicillin, and 100 μg/mL streptomycin and grown on sterile chambered coverglass (Labtek 8-well chambered #1 borosilicate coverglass, Thermo Scientific, Rochester, NY) maintained at 37 °C in humidified air (5% CO2) for 5-8 days prior to imaging. In other experiments, sections of posterior cerebral arteries were cannulated and pressurized in an arteriograph (CH-1 single vessel chamber, Living Systems Instrumentation, St Albans, VT) then loaded with, and imaged in, TMRE (62.5 nM at 36 °C) in the vessel’s bathing solution.

### Imaging and image processing

TMRE was illuminated at 555 ± 5 nm (xenon arc lamp output selected by a monchromator, PTI Inc.) and emitted light >580 nm imaged by a Photometrics Cascade 512B camera (Roper Scientific, controlled by WinFluor v3.4.5) at 10 Hz (100 ms exposure). While the TMRE image shows mitochondria within the cell, fluorescence overlap derived from organelles that are close to each other obscures boundaries and images of individual mitochondria. The method of recovery of the images of each mitochondrion is described below.

The individual image (fluorescence, F) for each mitochondrion (i) may be considered as: F_i_(x, y, t) = F_spatial_,_i_(x, y)*F_temporal_,_i_(t)*K_i_, where F_spatial_ is the conventional fluorescence image that would result from imaging only those fluorophores contained within the i^th^ organelle (normalized to between 0 and 1), over the Cartesian pixel grid (x, y). F_temporal_ is the relative fluorescence intensity at time t (dictated by the mitochondrion’s polarization state at that time). F_temporal_ changes over time as a result of changes in mitochondrial membrane potential. K_i_ is an intensity scaling constant derived from variables which include fluorophore concentration, maximum mitochondrial membrane potential, organelle dimensions, plasma membrane potential etc. Where two mitochondria (i and j) are close, F_spatial_ from each organelle overlaps (Fig. 1b), and an “area of confusion” (AoC) exists between them so that their separate identities cannot be resolved because F_spatial_,_i_ and F_spatial_,_j_ are mixed. On the other hand, away from the AoC, the fluorescence intensity signals recorded are straightforward and proportional to the respective mitochondrion’s F_spatial_ and F_temporal_ (example F_temporal_,_i_ and F_temporal_,_j_ are shown in Fig. 1b′).

F′_i_ (the time derivative of the fluorescence intensity signal, shown in Fig. 1b’ as dI/dt) spikes only briefly at the onset and termination of mitochondrial depolarization periods (flickers) and was used to separate individual organelle identities. Since mitochondrial de- and re-polarizations at the onset and termination of flickers are rapid when compared to the duration of the imaging period, the probability of two adjacent mitochondria de- or re-polarizing simultaneously tends to zero, with the consequence that the covariance of the time derivative of their respective F_temporal_ signals is 0, i.e. COV(F′_temporal,i_, F′_temporal,j_) = 0 when i ≠ j. As such, F′_temporal,i_ offers a unique signature of each mitochondrion (see also^23^,^26^). By selecting a candidate pixel (x_a_, y_a_) which is clearly inside mitochondrion M_i_ and thus away from any AoC, the fluorescence intensity measured is proportional to that mitochondrion’s F_temporal_,_i_ signature. Measuring the covariance of this signature with F′(x, y) for every pixel (x, y) in the vicinity of the candidate (COV(F′_i_(x_a_, y_a_), F′_i_(x, y)) generates a spatial covariance map that may be shown to be proportional to F_spatial_,_i_ and the variance of the fluorescence intensity over time, i.e. COV(F′(x_a_, y_a_), F′(x, y)) α F_spatial,i_(x, y) * VAR(F′_temporal,i_). A knowledge of VAR(F′_temporal,i_) is not required to recover F_spatial,i_(x, y) i.e. the relative intensity image of the mitochondrion. Importantly, this approach (FaLM) will separate individual mitochondria, irrespective of how many organelles are neighbors or on which axis the overlapping mitochondria lie.

The operator interactively explores the data by generating covariance maps for manually selected candidate pixels, resolving areas of confusion and with suitable candidate pixels being recorded in the software as organelle centers. A covariance map is then generated for each organelle center, representing F_spatial_ maps for each mitochondrion, which can be recombined and false-colored for visual inspection (Figs 1e and 2a’) or analyzed further (Figs 4 and 5). An additional pre-processing step was employed to compensate for baseline drift caused by positional and intensity changes, this is fully described below. The individual steps of the full pipeline and example output data are shown in Fig. S1*A* and *B* respectively.

### FaLM Algorithm

The rate of change of fluorescence, after some normalization, represents the de- and re-polarization events of individual mitochondria, which may be used as organelle-unique fingerprints to determine the spatial extent of individual mitochondria. We start with an intensity cube of I(x, y, t) where xy are pixel coordinates and t is frame number.

**Pass 1-** “all_on”. To accommodate gradual baseline drift caused by photo-bleaching, time varying dye distribution and motion (primarily gradual creep caused by minor cell conformational change) we calculate, at each time point, what the image would look like if all mitochondria were fluorescing. The time window selected is short enough to localize baseline drift, but long enough to guarantee that every organelle is, at some point in that window, polarized and fluorescing. The “all_on” image is generated by, on a per pixel basis, taking the average of the brightest 20% of intensities from the bracketing range or 1200 frames (120 s, Fig. S1Ai).

**Pass 2**–“Delta”–the Delta image is calculated by subtracting each frame of the original image stack from its corresponding “all_on” image–D(x, y, t) = all_on(x, y, t) – I(x, y, t). This process visualizes only mitochondria that are currently depolarized (Fig. S1Aii). In this processing step, during a depolarization event of a specific mitochondrion, all pixels associated with the mitochondrion simultaneously become bright. Individual mitochondria could therefore be located manually at the times in which they are depolarized, and then their spatial properties be extracted, however this would be a time consuming process involving frame-by-frame examination of the data. Measurement of the covariance of the temporal signal between spatial pixels could be used, but there exists a high probability of near-by mitochondria having some periods of coincident depolarization, leading to cross talk in the analysis. To overcome this, the signals were differentiated.

**Pass 3**–“dD/dt”. This signal is the time-derivative of the Delta signal (Fig. S1Aiii), such that the effect of a period of depolarization is reduced to brief spikes during the actual de- and re-polarization events themselves, which are much briefer in duration that the period of depolarization. This brevity reduces the probability of collisions in the signals sufficiently to allow the signal to be used for a covariance analysis. Whilst the raw data could have been directly time differentiated instead of the Delta signal, the resultant images would have been subject to baseline drift causing spurious covariance.

**Pass 4**–covariance analysis–the operator estimates the center of each mitochondrion in the image. This is done by picking a candidate center, for which the software plots the covariance of that pixels’ dD/dt with the dD/dt of neighboring pixels, denoting the extent of that candidate’s signal. In this way a dense clump is rapidly explored, with centers being identified and recorded (Fig. S1Aiv). Finally all recorded centers are used to fully identify all the individual, electrically-discrete mitochondria within the cell (Fig. S1Av).

### Mitochondrial Clustering

To perform quantitative analyses, two measures of relative mitochondrial positioning were developed. For each mitochondrion we consider a) the number of mitochondrial neighbors and b) the average distance to those neighbors. Where there exists a point equidistant to two mitochondria, and for which no other mitochondrion is closer, those two mitochondria are considered neighbors. To achieve this, a Voronoi diagram was constructed. Voronoi diagrams provide a quantitative method to determine which mitochondria are closest neighbors, the number of neighbors each organelle has and the distances between neighbors. The Voronoi plot does this by partitioning the biological cell into many regions, one per mitochondrion, where each region (called a ‘Voronoi cell’) represents the part of the smooth muscle cell closest to its mitochondrion. The Voronoi diagram was generated around nodes located at the center of each mitochondrion (defined by the center of mass of their respective FaLM binary masks), creating a series of Voronoi cells. Each Voronoi cell contains one mitochondrion and all points closer to that mitochondrion than any other. Each edge in the Voronoi diagram is shared between two Voronoi cells, and represents all points equipoised to the mitochondria in both Voronoi cells. By automatically creating and parsing the diagram, a list of neighbor pairs is created, allowing automated measurement of neighbor number and distance. This was further refined as some generated Voronoi cells, extend outside the physical cell boundaries, with distant mitochondria therefore being declared neighbors because of a shared edge that was outside the extent of the cell. An additional requirement was added: for two mitochondria to be declared neighbors, their Voronoi cells must be joined by an edge that falls inside the outline of the biological cell (grey area, Fig. 5c). This outline was generated by manual tracing of the associated cell’s brightfield images.

### Model Flickering Fluorescent Objects

The ability of FaLM to detect flickering objects was assessed by creating model image stacks of 18 stochastically-flickering fluorescent objects (1.28–8.13 μm^2^) blurred by a realistic point spread function (480 nm FWHM) plus simulated photobleaching (exponential decay to 67% of original intensity over the duration of a 120 s, 10 Hz image stack), a simulated gain of 1 ADU/photoelectron, read noise (Gaussian distribution, 3e^−^ FWHM) and photon (shot) noise, resulting in 5 image stacks with decreasing signal to noise ratio (SNR) of 28.13, 13.83, 6.52, 4.29 and 2.62, measured at the center of an object. Objects underwent transient flickers, randomly distributed after the initial 15 s, with an average of 4.1 flickers per object. Flicker durations were assigned at random to a uniform distribution between 1.5 s and 5 s.

### 3D Imaging

For z-stack imaging (data presented in Supplementary Video 1) an additional 1.5x lens within the microscope was introduced into the light path (decreasing pixel width from 225 to 150 nm). 250 image slices were taken at 100 nm intervals and XYZ data deconvolved in Image Pro Analyzer 3D (v 7.0.1.658, Media Cybernetics Inc, Rockville, MD) using its SharpStack 3D blind deconvolution set to 60 iterations with auto noise reduction and emission wavelength of 584 nm.

### Statistics

Data were tested for normality prior to statistical testing. Mitochondrial area, number per cell, length, width, length:width ratio, cell area and proportion of the cell occupied by mitochondria were not normally distributed and are thus described using median values and the interquartile range (IQR) for *N* animals within each strain (SHR and WKY). Statistical differences between the median values for each strain were calculated by a two-sample Kolmogorov-Smirnov test for non-parametric data using OriginPro 9.0.0 (OriginLab, Northampton, MA), with significance set at *α* = 0.05 and actual *p*-values quoted throughout. The number of neighboring mitochondria and intermitochondrial distances were normally distributed and are hence described using mean values ± standard error of the mean (SEM), statistical differences calculated by Student’s t-test with Welch’s correction of *p* value for unequal variance using OriginPro 9.0.0.

## Additional Information

**How to cite this article**: Chalmers, S. *et al.* Flicker-assisted localization microscopy reveals altered mitochondrial architecture in hypertension. *Sci. Rep.* **5**, 16875; doi: 10.1038/srep16875 (2015).

## Supplementary Material

Supplementary Information

Supplementary Video 1

Supplementary Video 2
