# Supplementary material for: Flicker-assisted localization microscopy reveals altered mitochondrial architecture in hypertension
Source: Sci Rep. 2015 Nov 23;5:16875. doi: 10.1038/srep16875 (PMC4655370; doi:10.1038/srep16875)

Title: Flicker-assisted localization microscopy reveals altered mitochondrial architecture in hypertension

Susan Chalmers[1], Christopher Saunter[2], John M. Girkin[2], John G. McCarron[1*]:

[1]Strathclyde Institute of Pharmacy & Biomedical Sciences, 161 Cathedral Street, University of Strathclyde, Glasgow G4 0RE, UK;

[2]Department of Physics, Durham University, South Road, Durham DH1 3LE, UK

Correspondence should be addressed to J.G.M. (email: john.mccarron@strath.ac.uk; tel: +44 141 548 4119).

Supplementary Information

Figure Legends

Supplementary Video 1 | 3D projection of mitochondria within a freshly-isolated resistance artery smooth muscle cell. The live cell was loaded with TMRE (62.5 nM) and epifluorescence images taken with increasing z-depth (250 x 100 nm steps). The images were then deconvolved using a 60-iteration blind 3D-deconvolution as described in Methods. The resulting image stack was rotated in the y-axis first at full 512x512 pixel/76.8x76.8 µm x-y magnification, followed by a 2x then a 5.2x zoom to show mitochondrial architecture.

Supplementary Video 2 | Mitochondrial flickering and localized fluorescence intensity changes detected by FaLM processing. A freshly-isolated smooth muscle cell loaded with TMRE (62.5 nM) and imaged by convention XYT epifluorescence displays localized fluctuations in fluorescence as individual mitochondria transiently de- and re-polarize (upper panels, right-hand box is close up of the whole cell shown in the left-hand box). The image stack was then processed by FaLM and the resultant *dI/dt* stack overlaid to show depolarizations in red and repolarizations in blue (lower panels). Scale bars 10 µm.

Supplementary Figure 1 | Full description of FaLM image processing. A: FaLM processing of the TMRE image stacks was a multi-step process. The first pass (i) created an image stack (*all_on*) in which all pixels were projected at their maximum intensity within a broad bracketing range to accommodate movement artefacts. ii: Each plane of the original image stack (*f(t)*) was then subtracted from the time-matched plane from *all_on* to create a stack of difference from maximum (*Delta*). The third pass (iii) looked for changes within *Delta* to create a stack showing depolarizations as bright spots and repolarizations as dark holes (*dD/dt*). These on and off events were then recombined to provide a

guide for the user to locate mitochondria. The fourth pass (iv) analyzed the temporal and spatial covariance of the *dD/dt* events to define mitochondrial *objects*, which were then be recombined (v) to provide an image of all individual mitochondrial units (vi) or intensity-thresholded to define boundaries (vii) and allow spatial measurements. B: example images of a resistance artery smooth muscle cell taken through FaLM processing, whole cell in larger images (i and below) and close-up of networked-like region in smaller images (ii and below) with corresponding intensity plots from single point (iii and below).

Supplementary Video 1

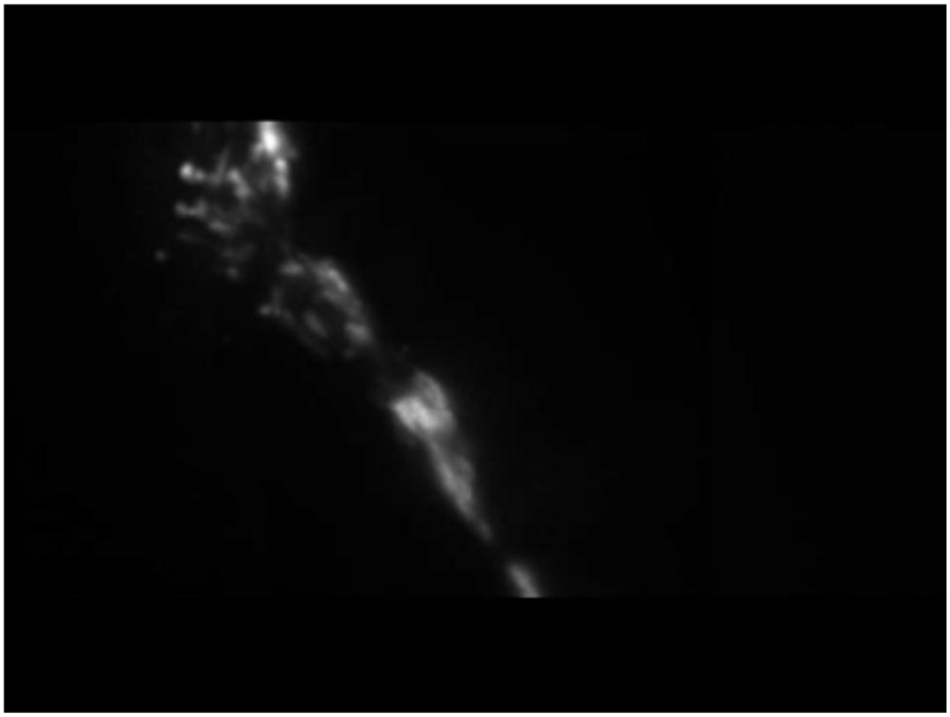

Supplementary Video 2

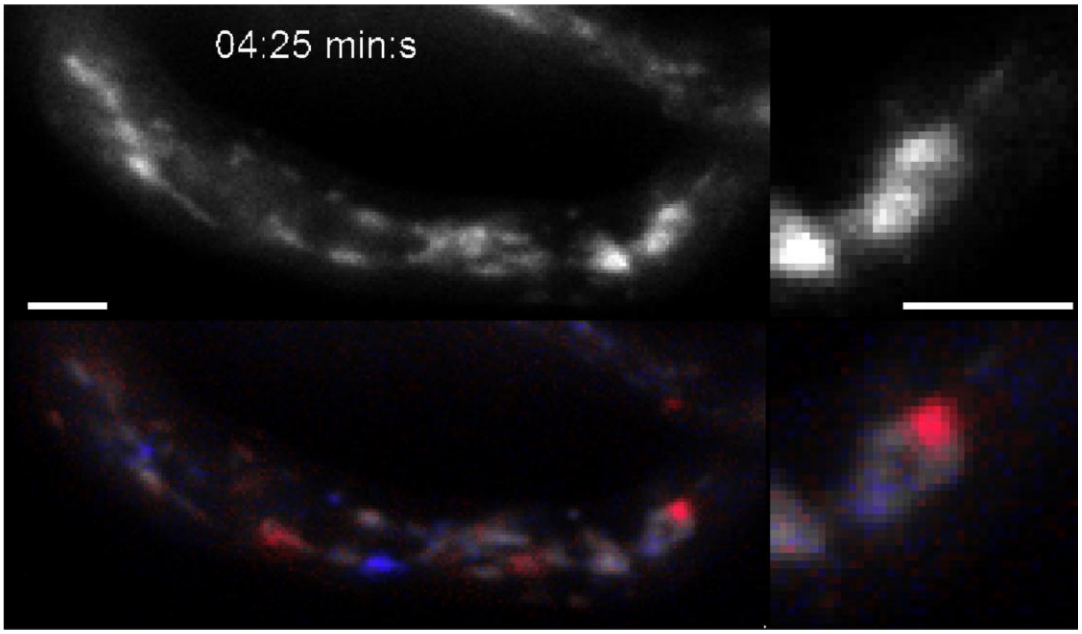

Supplementary Figure 1

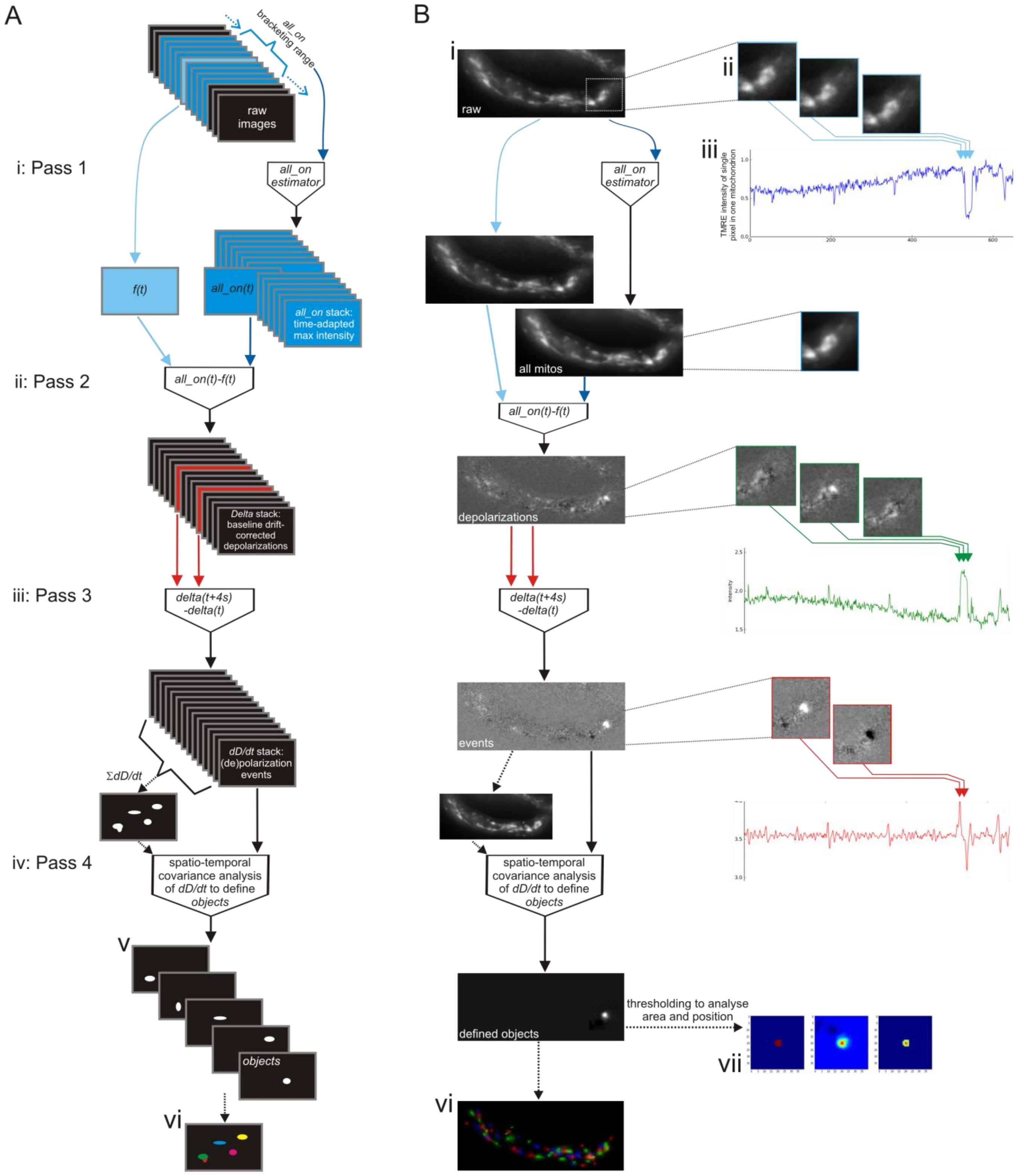

Supplement: Supplementary Information [file srep16875-s1.pdf]
